# A traditional Chinese medicine versus Western combination therapy in the treatment of rheumatoid arthritis: two-stage study protocol for a randomized controlled trial

**DOI:** 10.1186/1745-6215-12-137

**Published:** 2011-06-04

**Authors:** Chi Zhang, Miao Jiang, Aiping Lu

**Affiliations:** 1 Institute of Basic Research in Clinical Medicine, China Academy of Chinese Medical Sciences, Beijing, China

## Abstract

**Background:**

The common randomized controlled trial design has distinct limitations when applied to Chinese medicine, because Chinese medicine identifies and treats 'Chinese medicine patterns' rather than diagnosed diseases. Chinese medicine patterns are a group of associated symptoms, tongue appearances and pulse characteristics. These limitations could be overcome by developing new strategies to evaluate the effect of Chinese medicine. The idea behind pattern-based efficacy evaluations may optimize clinical trial design by identifying the responsiveness-related Chinese medicine patterns.

**Methods/Design:**

This is a two-stage multi-center trial of Chinese herbal medicine for the management of rheumatoid arthritis. The stage one trial is an open-label trial and aims to explore what groups of Chinese medicine information (such as symptoms) correlates with better efficacy, and the stage two trial is a randomized, controlled, double-blind, double-dummy clinical trial that incorporates the efficacy-related information identified in the stage-one trial into the inclusion criteria.

**Discussion:**

The indication of a Chinese herbal formula is a specific Chinese medicine pattern and not a single disease and stratifying a disease into several patterns with a group of symptoms is a feasible procedure in clinical trials. This study is the first to investigate whether this approach in the design of Chinese herbal medicine trials can improve responses.

**Trial registration:**

ChiCTR-TRC-10000989

## Background

Clinical trials are mainly aimed at showing the efficacy and safety of a therapy after initial indications are determined in preclinical studies, but the results obtained from a clinical trial have become less important in identifying the specific indications of a therapy. At present, "-omics"-based pharmacology contributes to individualized medicine, which attempts to find more specific indications for therapies by identifying biomarkers [1]. Similarly, Chinese medicine (CM) generally prescribes therapies based on the identification of CM patterns in the patient by analyzing the symptoms and characteristics [2].

All medicines should be rigorously tested [3,4], and the WHO Traditional Medicine Strategy suggests that clinical trials should be performed to establish the efficacy of CM [5]. Randomized clinical trials are useful to evaluate the efficacy of single drugs or combination therapy in a biomedical context [6]. However, they have distinct limitations when applied to CM because CM treatments focus on CM pattern classification [7]. Thus, alternative strategies, based on the CM concept, must be developed for the evaluation of CM efficacy and safety [8].

One important terminology in CM is "one formula based on one pattern", and the concept behind CM pattern-based individualized medication is to find out the responsiveness-related biocharacteristics for the interventions [9]. Thus, in CM practice, patients with rheumatoid arthritis (RA) can be treated with different therapies according their different patterns, which are a group of symptoms [10]. Therefore, the key factor in evaluating the efficacy of herbal therapy is to find out the specific CM pattern or indication for herbal therapy. Although CM pattern classification is complicated and difficult to understand in terms of making individualized herbal combinations for patients, CM patterns for specified herbal formulae could be identified by finding out the responsiveness-related CM information. Thus, it is reasonable to hypothesize that the CM pattern for one herbal therapy could be determined by comparing the differences of CM information between the responsive and non-responsive patients treated with the herbal therapy.

To test this hypothesis, we propose a two-stage clinical trial sponsored by the Institute of Basic Research in Clinical Medicine, China Academy of Chinese Medical Sciences in China. The primary objective of the trial was to find out the responsiveness-related CM symptoms that could be used as criteria for inclusion into secondary clinical trials. By comparing the difference in CM information between the responsive and non-responsive cases treated with one specific herbal intervention, the efficacy related information could be identified by statistical approaches. Secondary trials would then be aimed at evaluating the clinical efficacy for the herbal therapy by incorporating the efficacy related CM information into the inclusion criteria.

## Introduction to interventions

### CM interventions

The combination of Chinese herbal drugs Yi Shen Juan Bi (YSJB) and Tripterygium Wilfordii Polyglycoside (both drugs in tablet form) are approved as herbal medications by the State Food and Drug Administration (China) in patients with RA. Combination therapy is recommended by the Chinese Association of Integrative Medicine (unpublished data). The YSJB formula has been recognized as a valuable CM used in the treatment of RA. Clinically, YSJB has been shown to ameliorate symptoms and signs of RA and to decrease the erythrocyte sedimentation rate (ESR), C-reactive protein (CRP), and the rheumatic factor (RF) [11]. Additionally, the mechanisms underlying the efficacy of YSJB have been studied; YSJB significantly decreased the production of peritoneal macrophage-derived TNF-α, IL-1 and NO and significantly decreased prostaglandin E (PGE) and up-regulated Bax expression in rat synovium [12-14]. Components of YSJB are shown in Table 1. Tripterygium Wilfordii Polyglycoside (TWP, dark brown pills, patent number: Z34021048) is a commonly used anti-rheumatic herbal drug. In CM, extracts of Tripterygium wilfordii Hook F (TwHF, known as "lei gong teng" or "thunder god vine") are used to treat autoimmune and inflammatory conditions. Clinical trials suggest that TwHF may benefit patients with RA [15,16].

**Table 1:** Components of Yi Shen Juan Bi (YSJB)

Chinese name	Latin name
Cang Er Zi	Fructus Xanthii
Congrong	Herba Cistanchis
Dang Gui	Radix angelica
Di Huang	Radix rehmanniae
Di Long	Pheretima
Gan Cao	Radix Glycyrrhizae
Gu Bus Ui	Rhizoma drynariae
Hu Zhang	Polygoni cuspidati
Ji Xue Teng	Caulis spatholobi
Jiang Can	Bombyx batryticatus
Lao Guan Cao	Herba erodii
Lu Xian Cao	Herba pyrolae
Qiang Lang Chong	Allomyrina dichotoma
Quan Xie	Scorpio
Shu Di Huang	Radix rehmanniae praeparata
Tu Bie Chong	Eupolyphaga seu steleophaga
Wu Gong	Scolopendra
Wu Shao She	Zaocys (stir-fried with wine)
Xu Chang Qing	Cynanchi paniculati
Xun Gu Feng	Herba Aristolochiae
Yan Hu Suo	Rhizoma corydalis
Yin Yang Huo	Herba epimedii
Zhi Feng Fang	Nidus vespae (stir-bakedg)
Zhi Feng Fang	Nidus Vespae

### Control interventions

The combination of methotrexate (MTX) and sulfasalazine (SSZ) is also a commonly used therapy for RA treatment [17], and the combination is used as a control in this clinical trial.

### Design

This is a two-stage, multi-center trial in the management of RA. The first-stage trial is an open-label trial and aims to explore which groups of CM-related symptoms correlates with better efficacy, and the second-stage trial is a 24-week randomized, controlled, double-blind, double-dummy clinical trial that incorporates the group of symptoms identified in the first-round trial in the inclusion criteria.

The hypotheses for the two-stage clinical trial to be tested are as follows:

1. The CM therapy is safe, acceptable and feasible, and at least 80% of the subjects will complete the CM therapy.

2. The specified group of participants treated with CM therapy will demonstrate significantly improved ACR20 responses compared to the positive control group.

**Figure 1:**
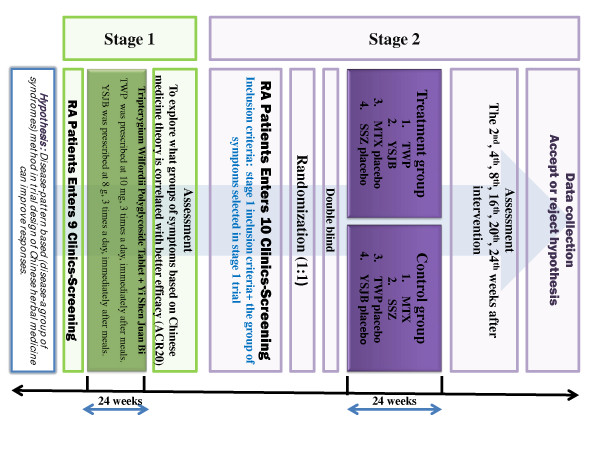
**The flowchart of the two-stage trial design**.

A flow chart of the study design is shown in Figure 1.

## Stage one of the Trial

The first-stage trial is a 24-week open-label trial and is aimed at exploring what CM information correlates with better efficacy of the herbal intervention. The sample size is 120 cases.

### Inclusion criteria for participants

Eligible patients should be ≥ 18 and ≤ 70 years old and should have been diagnosed with RA according to American College of Rheumatology (ACR) classification criteria [18] and have active RA, which is defined as class I, II, or III RA, at the time of screening [19]. Patients are allowed to take stable doses of non-steroidal anti-inflammatory drugs or oral corticosteroids (≤10 mg prednisone or equivalent) ≥ 4 weeks before screening. Signed, informed consent from a patient or guardian will be required.

### Exclusion criteria for participants

Patients were excluded from the study if they received other commercial or experimental biological therapies for RA, were diagnosed with another inflammatory disease within one month of screening, or experienced uncontrolled or clinically significant systemic disease other than RA. Patients diagnosed with class IV RA as defined by ACR revised criteria for global functional status in RA or any major chronic inflammatory disease or connective tissue disease other than RA were also excluded. Patients would be ineligible if they had any laboratory abnormality that, in the opinion of the investigator, would prevent the patient from completing the study or interfere with the interpretation of the study results. Patients should not have received intra-articular or systemic corticosteroid injections or any investigational therapy within 4 weeks of screening, any disease-modifying anti-rheumatic drug other than MTX and SSZ within 4 weeks of screening, or cyclophosphamide within 4 weeks of the first dose of the investigational product. Patients would be ineligible if, in the investigator's opinion, they have any physical or psychiatric disorder that could interfere with their ability to give informed consent or participate in the study. Pregnant women would not be eligible for the study.

### Intervention

Tripterygium Wilfordii Polyglycoside tablets were provided by Jiangsu Meitong Co., Ltd (Z43020138). Tripterygium Wilfordii Polyglycoside or placebo were prescribed at 10 mg 3 times a day immediately after meals. Yi Shen Juan Bi (YSJB, dark brown pills, patent number: ZL200510040550) is prepared by Qingjiang Co., Ltd (Z10890004) and were prescribed at 1 pocket (8 g) 3 times a day immediately after meals.

### Outcome measures

The primary efficacy endpoint is the ACR20 response [20] at week 24. Secondary efficacy endpoints include the ACR50 and ACR70 responses and the individual components of the ACR. Patterns based on CM symptoms, joint function, the Disease Activity Score using the 28-joint count (DAS28), physical exam, laboratory tests, and patient-reported outcomes, including the Disability Index of the Health Assessment Questionnaire (HAQ) were also noted [21]. All of these endpoints over time in the study will be analyzed as exploratory endpoints. Safety endpoints include adverse events (AEs), serious AEs (SAEs), and laboratory abnormalities.

### Ethical considerations

The study was conducted according to the Declaration of Helsinki and the International Conference on Harmonization Tripartite Guideline on Good Clinical Practice [22]. Approvals from the appropriate research ethics committees were obtained at each participating study center. All patients were asked to provide written, informed consent before participating.

### Withdrawal criteria

Patients would be withdrawn from the study if the clinician found that they were in need of or were better served with another treatment or if the exclusion criteria were met. Patients would also be withdrawn from the study if according to the clinician's judgment clinical conditions worsened or if the patients withdrew their consent. The date and reason for discontinuation were noted. All patients who prematurely discontinue the trial would undergo a final evaluation.

### Recruitment

Patients were recruited through hospital advertisements, and patients with RA would be likely to receive Chinese herbal medicine therapies.

### Setting

The trials were performed in the following hospitals: China-Japan Friendship Hospital, Nantong Liangchun Rheumatism Hospital, Xiangya Hospital affiliated with Zhongnan University, Traditional Chinese Medical Hospital of Xinjiang Uygur Autonomous Region, Traditional Chinese Medical Hospital of Anhui Province, Shanxi Taiyuan Rheumatism Hospital, Tianjin Chinese Medicine College-Affiliated First Hospital, and Tongji Hospital affiliated with Tongji Medical College, Huazhong University of Sciences and Technology.

### Statistical analysis and primary results

Patients were analyzed according to the open-label study. The primary efficacy endpoint compared the ACR20 response rate at week 24. All secondary endpoints were pre-specified for sequential testing. The correlation between the CM symptoms and the response, T-tests and correlation analyses were used for the statistical analysis. For dichotomous variables, missing values were imputed using a non-responder imputation method, and for continuous variables, the primary analysis were based on observed cases. Our primary result shows that the patients with large amounts of clear urine, who spontaneous sweated and experienced night sweats were found to be positively correlated with the ACR 20 response. Further analysis shows that the ACR20 for the CM intervention is 76% if only the RA patients with the efficacy related CM symptoms could be treated with the CM intervention, though the ACR20 is 58% in all RA patients.

## Stage two of the Trial

The investigation will be a double-blind, double-dummy, controlled study in patients with active RA aimed at exploring the efficacy of the herbal intervention in RA patients with specific CM symptoms. The secondary stage will be designed by adding the efficacy related CM symptoms obtained from the stage-one trial as the inclusion criteria.

### Inclusion criteria for participants

To be enrolled, patients will have to meet the inclusion criteria of stage one and have to show the efficacy related CM symptoms from the first-stage trial. At moment, large amounts of clear urine, spontaneous sweated and experienced night sweats were found to be efficacy related symptoms, and should be included in the inclusion criteria. More analysis is still conducted and hopefully we could find more positive clinical manifestations for the inclusion criteria.

### Exclusion criteria for participants

The exclusion criteria will be approximately the same as those of stage one. RA patients without the efficacy related CM symptoms from the first-stage trial will be excluded. Also we are conducting further analysis and trying to find the CM symptoms which are negatively related to the ACR20 response, and the patients with the efficacy negatively related CM symptoms will be excluded.

### Sample size

This trial at stage 2 is designed as an active control superiority trial and intended to show superiority of CM intervention. The sample size calculation is based on the ACR20 response rate of the stage one of the trial. The primary results of the stage one trial (76% ACR20 response of CM intervention in the patients with the efficacy positively related CM symptoms) and our previous clinical data (53% ACR20 response in Western medicine intervention in the patients with the efficacy positively related CM symptoms). The sample size was established by comparing the rates of effectiveness between CM and Western medicine interventions (set α = 0.05, *β*= 0.10). Sample size could be 88 in both groups. With a dropout rate for follow-up as 20%, the sample size for each group could be 110 and 220 in the two groups.

### Randomization

Randomization will be controlled by an independent clinical research coordinator (CRC). First, the randomization form with the basic information of the participant who passed the screening phase will be transmitted by facsimile to the independent statistician. The randomization number in the randomization form will be left blank before arrival at the statistician. The statistician will then decide the randomization number based on the allocation sequence, which will be generated by a random number creation program in advance. The statistician will then return the randomization form filled in with the established number (specific ID number) to the CRC. The ratio of randomization allocation to the sites should be 1:1. The CRC will then inform the investigators of the specific ID number. The completion of this procedure will be guaranteed by the research organization.

### Blinding

In this trial, investigators will not be in contact with the CRC, the clinical pharmacist, or the statistician. The CRC will be separated from all researchers, thus the researchers will not have any influence on enrollment or randomization. The statistician will receive the randomization form by facsimile, fill in the blank and return the form; thus, any contact with other researchers will not occur.

### Setting

In this stage, 2 or more centers will be added to the list of centers participating in the stage-one trial.

### Interventions

1. Tripterygium Wilfordii Polyglycoside and its associated placebo. To completely blind the patients, the Tripterygium Wilfordii Polyglycoside tablet and placebo are packaged identically. Either Tripterygium Wilfordii Polyglycoside or the placebo will be prescribed at 10 mg 3 times a day immediately after meals.

2. Yi Shen Juan Bi (YSJB) and its associated placebo. YSJB and its placebo are made by Qingjiang Pharmaceutical Co., Ltd (Z10890004). The placebo, which has an identical appearance, is made from cane sugar and will be prescribed at the same dosage as the YSJB, which will be 1 pocket (8 g) 3 times a day immediately after meals.

3. Methotrexate (MTX) and its associated placebo. Methotrexate tablets and the placebo will be provided by Shanghai Xinyi Pharmaceutical Co., Ltd (Drug registration Number: Z070404). MTX and its placebo are available in 2.5-mg tablets and will be administered orally at an initial dose of 5 mg/week (up to 15 mg/week, 2.5 mg/per time). For the remission period, the standard dose will range from 1 tablet/week (2.5 mg) to 3 tablets/week (7.5 mg).

4. Sulfasalazine (SSZ) and its associated placebo. Sulfasalazine tablets and the placebo were provided by Xi'an Kangbaier Pharmaceutical Co., Ltd (Drug registration Number: Z070404). SSZ and its placebo are available in 0.25 g tablets and will be taken at an initial dose of 0.25 g twice a day (up to 0.5-1 g, 4 times/day). For the remission period, the standard dose will be 0.5 g (2 tablets) 3-4 times/day.

Participants in the CM therapy will be given Tripterygium Wilfordii Polyglycoside and YSJB along with the MTX and SSZ placebos. Participants in the positive control therapy will be given MTX and SSZ (Western combination therapy) along with the Tripterygium Wilfordii Polyglycoside and YSJB placebos.

### Outcome measures

The primary efficacy endpoint is the ACR20 response. Secondary efficacy endpoints include the ACR50 and ACR70 responses and the individual components of the ACR. CM symptoms, joint function, physical exam, laboratory tests, and HAQ will be tested. Safety endpoints include AEs and laboratory abnormalities. The timeline of the participants' progression through the trial in stage two is shown in Table 2.

**Table 2:** Timeline of participants' progression through the stage-two trial

Assessment	Visit 1	Visit 2	Visit 3	Visit 4	Visit 5	Visit 6	Visit 7	Visit 8	Visit 9
	Screen	Base	2^nd ^w*	4^th ^w	8^th ^w	12^th ^w	16^th ^w	20^th ^w	24^th ^w (or withdrew)
Medical history	●								
Physical examination	●		●	●	●	●	●	●	●
Joint function		●	●	●	●	●	●	●	●
Swollen joint count		●	●	●	●	●	●	●	●
Tender joint count		●	●	●	●	●	●	●	●
Physician global status	●	●	●	●	●	●	●	●	●
Patient global assessment		●	●	●	●	●	●	●	●
Morning stiffness		●	●	●	●	●	●	●	●
Pain at rest		●	●	●	●	●	●	●	●
Pattern based on CM (symptoms)	●	●	●	●	●	●	●	●	●
Blood test		●	●	●		●			●
Routine urine test		●	●	●		●			●
Stool routine test		●				●			●
CRP		●				●			●
ESR		●				●			●
RF		●				●			●
ALT		●	●	●		●			●
AST		●	●	●		●			●
Bilirubin		●				●			●
Cr		●	●	●		●			●
BUN		●	●	●		●			●
VAS		●		●	●	●	●	●	●
HAQ		●				●			●
EGG		●							●
X-ray		●							●
Adverse events		●	●	●	●	●	●	●	●

Notes: *w = week

### Ethical considerations

Approvals from the appropriate research ethics committees will be obtained at ten study centers. All patients will be asked to provide written, informed consent before participating.

### Quality control

The identification, registration and subsequent flow of participants in the trial will be governed by the trial standard operational protocol (SOP) at all sites. The sites' Case Report Form (CRF) completion and compliance with standard operation procedures will be audited. Clinical research associates, at regular periods, will monitor the clinical trial procedures, such as compliance with administration and voluntary withdrawal of participants. In particular, the reasons for withdrawal will be fully documented in the CRF.

### Statistical analysis

Patients will be included for analysis according to the randomized treatment arm regardless of the actual treatment received during the study. All efficacy endpoints will be analyzed using the intent-to-treat analysis set, which will include all randomized patients regardless of whether they received an investigational product. The safety dataset will also include all patients who received ≥ 1 dose of an investigational product. The primary efficacy endpoint will be used to compare the ACR20 response rate at week 24 in the CM group with that of the biomedicine group. All secondary endpoints will be tested sequentially in a pre-specified order to control the one-error rate at 5% (two-sided). The comparisons of proportions (for dichotomous variables) among treatment arms will be performed using Fisher's exact test. For dichotomous variables, missing values will be imputed using a non-responder imputation method; for continuous variables, the primary analysis will be based on observed cases.

## Discussion

In the application of Chinese herbal medicine, many systematic reviews report that it is difficult to show rigorous evidence for the effectiveness of herbal medicine [23,24], even though Chinese herbal medicine has been used for over 3000 years in China and its effectiveness is based on thousands of years of observation and clinical practice. The reasons for this difficulty might be that the indication of a Chinese herbal medicine requires specification with CM pattern differentiation, which means that a Chinese herbal medicine should show its best effectiveness only in part of a population of patients with a specific disease.

The specific subpopulation of the patients could be identified with CM pattern classification principles. However, there are two questions in applying pattern classification to identify this subpopulation of patients. Firstly, the CM information for pattern classification, such as CM symptoms, the appearance of the tongue and the pulse, is not focused on biomedicine or is difficult for many biomedical practitioners to understand. Secondly, though the herbal products or a fixed herbal formula for efficacy evaluation would have its relative fixed CM pattern, it is still difficult to specify the exact CM pattern and identify the CM information for the pattern. In real CM clinical practice, the herbal preparations could be changed with additions or subtractions of the previous herbal combinations, and the changes in the herbal preparation are mainly based on the responses after the intervention. In that sense, the main concept in CM pattern identification would be that the specific CM pattern for an intervention could be determined as effectiveness-related factors by comparing the responsive and non-responsive cases based on the CM information. The effectiveness-related factors could be obtained by comparing the differences between the effective cases and non-effective cases if there was enough information in the clinical trial. Many non-diagnostic-related symptoms on which Chinese medicine focuses (such as thirst in rheumatoid arthritis or heavy limbs in diabetes), non-diagnostic laboratory measurements, and pharmaco-omics information used to determine the individual therapy can be collected for the comparison between the responsive and non-responsive cases [25,26]. At present, more attention is focused on "-omics" information for individualized therapy, and we believe that TCM information, with its effectiveness in long-term clinical practice, could be more feasible for clinical trials. Our previous analysis of a randomized clinical trial on an herbal product for the treatment of rheumatoid arthritis shows that some non-diagnostic symptoms correlated with the effectiveness of the drugs and that the effectiveness would be higher if the correlated information were re-analyzed [27].

The ingredients in herbal products, with their relative, fixed-CM pattern feature, cannot be changed after the product is marketed. Thus, there is a dilemma in evaluating the efficacy of CM herbal products; the CM pattern is important for the evaluation, but it is difficult to understand. This two-stage clinical trial was designed to evaluate the efficacy of CM therapy (biomedical intervention could be included in terms of indication specification) based on the CM pattern identification concept, and the CM patterns could be identified by comparing the CM information between the responsive and non-responsive cases during the first-stage clinical trial.

YSJB and TWP are commonly used for the treatment of RA in CM, and the combination therapy with MTX and SSZ are also commonly used in biomedicine. In routine clinical trials, YSJB and TWP show less effective in RA treatment compared to biomedical intervention (unpublished data) because it is hard to incorporate CM pattern information in trials. The findings from the stage-one trial suggested that the group of patients with some CM symptoms have improved efficacy, and the stage-two clinical trial was designed by adding the group of symptoms selected in the first stage trial for inclusion criteria in the second trial. We hope that the ACR20 response can be improved in RA treatment with CM therapy due to the CM pattern classification concept utilized in the trial.

It is anticipated that this two-stage trial will take 24 months to complete. The first-stage trial is completed, and recruitment into the second-stage trial commenced in June 2010 with the first participant randomized in August 2010. The 24-week follow-up assessments will be completed in the end of 2011.

## Abbreviations

ACR: American College of Rheumatology; AE: adverse event; CM: Chinese medicine; CRF: Case Report Form; CRP: C-reactive protein; ESR: erythrocyte sedimentation rate; MTX: Methotrexate; RA: rheumatoid arthritis; RCT: randomized controlled trial; RF: rheumatoid factor; SAE: serious adverse event; SOP: standard operational protocol; SSZ: Sulfasalazine; TWP: Tripterygium Wilfordii Polyglycoside; YSJB: Yi Shen Juan Bi

## Competing interests

The authors declare that they have no competing interests.

## Authors' contributions

CZ participated in the trial design and drafted the paper. MJ designed the trial and were responsible for the development of the protocol. AL and MJ are project leaders and have overall responsibility for the trial. All authors have read and approved the final manuscript.
